# Supplementary material for: Imputation-free transformer learning enables robust Alzheimer’s disease prediction and calibrated uncertainty quantification across heterogeneous clinical cohorts
Source: ArXiv. 2026 Jul 14:arXiv:2607.11656v2. Preprint. [Version 2] (PMC13405417)
Supplement: Supplement 1 [file NIHPP2607.11656v2-supplement-1.pdf]

Appendix A Supplementary information

Table A1: Performance of models trained on ADNI for CN vs. AD classification and evaluated on the ADNI train, validation, and test splits, as well as on the external OASIS-3 and AIBL cohorts. Models are sorted by AUC within each split. Seed = 123.

Data/Split	Model	Acc.	Bal. Acc.	Spec.	Sens.	F1	AUC
ADNI (Train)	XGBoost	1.000	1.000	1.000	1.000	1.000	1.000
	LightGBM	1.000	1.000	1.000	1.000	1.000	1.000
	MA-GBT	1.000	1.000	1.000	1.000	1.000	1.000
	Random Forest	0.986	0.985	0.994	0.975	0.984	0.999
	NITROGEN	0.914	0.908	0.952	0.863	0.896	0.972
	NAIM	0.917	0.912	0.951	0.873	0.901	0.969
	MA-Lasso	0.910	0.908	0.924	0.893	0.896	0.964
ADNI (Val)	NITROGEN	0.862	0.853	0.899	0.807	0.826	0.929
	XGBoost	0.856	0.853	0.870	0.835	0.825	0.925
	LightGBM	0.848	0.845	0.863	0.827	0.815	0.924
	NAIM	0.865	0.859	0.889	0.830	0.833	0.919
	MA-GBT	0.844	0.841	0.853	0.830	0.811	0.916
	MA-Lasso	0.854	0.850	0.868	0.832	0.822	0.910
	Random Forest	0.814	0.808	0.840	0.777	0.772	0.894
ADNI (Test)	LightGBM	0.854	0.846	0.875	0.817	0.807	0.925
	Random Forest	0.863	0.852	0.896	0.808	0.816	0.925
	MA-GBT	0.856	0.848	0.879	0.817	0.810	0.923
	XGBoost	0.861	0.854	0.881	0.827	0.816	0.922
	NITROGEN	0.846	0.841	0.862	0.821	0.800	0.915
	NAIM	0.826	0.824	0.831	0.817	0.779	0.912
	MA-Lasso	0.822	0.826	0.812	0.840	0.780	0.909
AIBL	NITROGEN	0.894	0.811	0.926	0.697	0.649	0.936
	MA-Lasso	0.902	0.840	0.926	0.755	0.684	0.931
	LightGBM	0.911	0.786	0.960	0.613	0.660	0.929
	XGBoost	0.917	0.782	0.970	0.594	0.669	0.923
	NAIM	0.905	0.812	0.941	0.684	0.669	0.919
	MA-GBT	0.913	0.806	0.955	0.658	0.680	0.917
	Random Forest	0.897	0.765	0.949	0.581	0.614	0.913
OASIS-3	MA-GBT	0.716	0.673	0.735	0.611	0.395	0.738
	NITROGEN	0.560	0.638	0.526	0.751	0.341	0.718
	NAIM	0.639	0.642	0.638	0.646	0.352	0.714
	XGBoost	0.542	0.630	0.504	0.756	0.334	0.714
	LightGBM	0.561	0.621	0.535	0.706	0.328	0.701
	MA-Lasso	0.572	0.622	0.550	0.693	0.329	0.697
	Random Forest	0.456	0.573	0.405	0.741	0.292	0.667

Table A2: Performance of models trained on ADNI for CN vs. AD classification on ADNI test set, AIBL, and OASIS-3 cohorts, reported as mean ± standard deviation across 10 random seeds (`seeds` = [123, 42, 0, 7, 13, 21, 37, 55, 77, 99]). Models are sorted by AUC within each split.

Data/Split	Model	Acc.	Bal. Acc.	Spec.	Sens.	F1	AUC
ADNI (Test)	LightGBM	0.869 ± 0.018	0.861 ± 0.020	0.895 ± 0.031	0.827 ± 0.041	0.829 ± 0.030	0.935 ± 0.014
	XGBoost	0.864 ± 0.019	0.857 ± 0.019	0.887 ± 0.032	0.827 ± 0.034	0.824 ± 0.030	0.933 ± 0.012
	NITROGEN	0.848 ± 0.023	0.845 ± 0.022	0.858 ± 0.038	0.832 ± 0.035	0.809 ± 0.032	0.924 ± 0.016
	Random Forest	0.851 ± 0.025	0.841 ± 0.025	0.888 ± 0.037	0.794 ± 0.039	0.806 ± 0.036	0.916 ± 0.020
	NAIM	0.846 ± 0.024	0.839 ± 0.024	0.870 ± 0.036	0.808 ± 0.037	0.802 ± 0.037	0.915 ± 0.019
	MA-Lasso	0.839 ± 0.024	0.837 ± 0.023	0.841 ± 0.038	0.833 ± 0.037	0.800 ± 0.035	0.913 ± 0.018
AIBL	XGBoost	0.917 ± 0.004	0.789 ± 0.012	0.967 ± 0.004	0.610 ± 0.025	0.674 ± 0.018	0.918 ± 0.005
	MA-Lasso	0.894 ± 0.011	0.838 ± 0.013	0.916 ± 0.014	0.761 ± 0.030	0.669 ± 0.024	0.917 ± 0.008
	LightGBM	0.911 ± 0.006	0.800 ± 0.014	0.954 ± 0.008	0.645 ± 0.031	0.670 ± 0.020	0.916 ± 0.008
	Random Forest	0.907 ± 0.002	0.771 ± 0.009	0.960 ± 0.005	0.582 ± 0.022	0.637 ± 0.009	0.913 ± 0.003
	NAIM	0.890 ± 0.010	0.809 ± 0.009	0.922 ± 0.017	0.695 ± 0.032	0.641 ± 0.014	0.905 ± 0.012
	NITROGEN	0.886 ± 0.020	0.807 ± 0.023	0.916 ± 0.033	0.697 ± 0.072	0.633 ± 0.026	0.901 ± 0.011
OASIS-3	LightGBM	0.613 ± 0.050	0.660 ± 0.032	0.593 ± 0.060	0.727 ± 0.036	0.365 ± 0.034	0.719 ± 0.020
	XGBoost	0.551 ± 0.074	0.641 ± 0.039	0.512 ± 0.090	0.770 ± 0.028	0.345 ± 0.036	0.713 ± 0.031
	NITROGEN	0.556 ± 0.060	0.636 ± 0.025	0.521 ± 0.078	0.751 ± 0.054	0.341 ± 0.023	0.710 ± 0.018
	NAIM	0.490 ± 0.056	0.602 ± 0.026	0.442 ± 0.071	0.763 ± 0.033	0.314 ± 0.021	0.703 ± 0.012
	Random Forest	0.610 ± 0.168	0.621 ± 0.048	0.606 ± 0.222	0.636 ± 0.140	0.349 ± 0.058	0.700 ± 0.031
	MA-Lasso	0.593 ± 0.021	0.616 ± 0.019	0.583 ± 0.029	0.650 ± 0.047	0.326 ± 0.016	0.689 ± 0.017

Table A3: Performance of models trained on ADNI for cognitively normal (CN) vs. cognitively impaired (MCI/AD) classification and evaluated across datasets. Models are sorted by AUC within each split. Seed = 123.

Data/Split	Model	Acc.	Bal. Acc.	Spec.	Sens.	F1	AUC
ADNI (Train)	XGBoost	1.000	1.000	1.000	1.000	1.000	1.000
	LightGBM	1.000	1.000	1.000	1.000	1.000	1.000
	MA-GBT	0.998	0.998	0.996	0.999	0.999	1.000
	Random Forest	0.992	0.987	0.974	1.000	0.994	1.000
	NAIM	0.825	0.773	0.640	0.906	0.878	0.880
	MA-Lasso	0.780	0.770	0.746	0.795	0.834	0.862
	NITROGEN	0.781	0.706	0.514	0.899	0.851	0.846
ADNI (Val)	LightGBM	0.744	0.677	0.459	0.894	0.821	0.810
	MA-GBT	0.721	0.655	0.442	0.868	0.803	0.795
	XGBoost	0.718	0.647	0.416	0.877	0.803	0.781
	Random Forest	0.700	0.584	0.214	0.955	0.806	0.769
	NITROGEN	0.703	0.633	0.409	0.857	0.791	0.761
	NAIM	0.702	0.646	0.467	0.826	0.784	0.749
	MA-Lasso	0.658	0.645	0.602	0.687	0.725	0.725
ADNI (Test)	MA-GBT	0.741	0.668	0.457	0.880	0.821	0.802
	LightGBM	0.744	0.672	0.463	0.881	0.822	0.799
	NITROGEN	0.727	0.648	0.418	0.877	0.812	0.794
	XGBoost	0.733	0.662	0.459	0.866	0.813	0.790
	Random Forest	0.706	0.581	0.221	0.942	0.812	0.785
	NAIM	0.731	0.668	0.488	0.849	0.809	0.781
	MA-Lasso	0.681	0.660	0.599	0.721	0.753	0.748
AIBL	Random Forest	0.400	0.575	0.206	0.944	0.454	0.766
	NITROGEN	0.453	0.591	0.300	0.882	0.460	0.757
	LightGBM	0.529	0.632	0.414	0.850	0.487	0.744
	MA-GBT	0.519	0.627	0.399	0.855	0.484	0.738
	XGBoost	0.570	0.636	0.496	0.776	0.487	0.733
	NAIM	0.510	0.622	0.385	0.858	0.480	0.732
	MA-Lasso	0.588	0.635	0.535	0.735	0.484	0.709
OASIS-3	LightGBM	0.288	0.545	0.159	0.931	0.304	0.700
	MA-Lasso	0.383	0.577	0.286	0.868	0.320	0.697
	NITROGEN	0.250	0.536	0.107	0.964	0.301	0.690
	Random Forest	0.215	0.516	0.064	0.969	0.292	0.668
	XGBoost	0.276	0.548	0.140	0.955	0.306	0.661
	MA-GBT	0.269	0.543	0.132	0.955	0.304	0.660
	NAIM	0.304	0.561	0.175	0.946	0.312	0.639

Table A4: Global classification performance of models trained on ADNI for three-class diagnostic classification (CN / MCI / AD), evaluated across datasets. ADNI comprised 7,858 samples divided into stratified 60/20/20 train/validation/test splits (Train: 4,704; Val: 1,561; Test: 1,593). OASIS-3 (n=2,675) and AIBL (n=1,286) were used as held-out external validation cohorts. Metrics reported are balanced accuracy (Bal. Acc.), macro-averaged F1, macro-averaged AUC under the one-vs-rest (OvR) scheme, Matthews Correlation Coefficient (MCC), and Cohen's κ. Macro-averaged metrics weight each class equally regardless of class frequency, which is appropriate given the class imbalance present across cohorts. Models are sorted by Macro AUC within each split. Seed = 123. Full per-class metrics are reported in Table A5.

Split	Model	Accuracy	Balanced Acc.	Macro F1	Macro AUC (OvR)	MCC	Cohen
ADNI (Train)	XGBoost	1.000	1.000	1.000	1.000	1.000	1.000
	LightGBM	1.000	1.000	1.000	1.000	1.000	1.000
	Random Forest	0.982	0.976	0.981	0.999	0.972	0.972
	NITROGEN	0.692	0.681	0.691	0.861	0.511	0.509
	NAIM	0.626	0.601	0.615	0.801	0.399	0.393
ADNI (Val)	XGBoost	0.582	0.580	0.589	0.782	0.342	0.339
	LightGBM	0.596	0.594	0.602	0.778	0.366	0.362
	Random Forest	0.570	0.550	0.557	0.765	0.326	0.304
	NITROGEN	0.541	0.543	0.552	0.737	0.277	0.276
	NAIM	0.523	0.519	0.526	0.719	0.247	0.242
ADNI (Test)	XGBoost	0.591	0.573	0.588	0.771	0.351	0.345
	LightGBM	0.588	0.579	0.590	0.762	0.350	0.347
	Random Forest	0.560	0.524	0.534	0.762	0.302	0.278
	NITROGEN	0.556	0.543	0.556	0.752	0.295	0.292
	NAIM	0.504	0.478	0.492	0.707	0.203	0.196
OASIS-3	LightGBM	0.265	0.383	0.247	0.596	0.117	0.062
	Random Forest	0.116	0.340	0.150	0.595	0.081	0.024
	NAIM	0.209	0.331	0.177	0.594	0.052	0.026
	NITROGEN	0.233	0.369	0.247	0.583	0.123	0.056
	XGBoost	0.206	0.339	0.209	0.574	0.091	0.044
AIBL	NAIM	0.394	0.479	0.410	0.729	0.195	0.127
	Random Forest	0.374	0.468	0.395	0.728	0.189	0.115
	LightGBM	0.467	0.492	0.454	0.726	0.209	0.158
	XGBoost	0.473	0.470	0.442	0.725	0.192	0.149
	NITROGEN	0.363	0.463	0.395	0.719	0.169	0.104

Table A5: Per-class classification performance (Recall or sensitivity, specificity, and AUC) for three-class diagnostic classification (CN / MCI / AD), evaluated across datasets. Sensitivity (Sens.) corresponds to the true positive rate for each class; specificity (Spec.) corresponds to the true negative rate. AUC is reported per class under the one-vs-rest (OvR) scheme. Models are sorted by Macro AUC within each split (same order as Table A4). Seed = 123. Global metrics are reported in Table A4.

		CN			MCI			AD		
Split	Model	Rec.	Spec.	AUC	Rec.	Spec.	AUC	Rec.	Spec.	AUC
ADNI (Train)	XGBoost	1.000	1.000	1.000	1.000	1.000	1.000	1.000	1.000	1.000
	LightGBM	1.000	1.000	1.000	1.000	1.000	1.000	1.000	1.000	1.000
	Random Forest	0.989	0.996	1.000	0.998	0.973	1.000	0.941	0.999	0.998
	NITROGEN	0.648	0.882	0.876	0.740	0.674	0.799	0.654	0.933	0.907
	NAIM	0.510	0.870	0.803	0.736	0.569	0.713	0.555	0.931	0.886
ADNI (Val)	XGBoost	0.496	0.828	0.798	0.635	0.572	0.670	0.609	0.918	0.877
	LightGBM	0.498	0.848	0.802	0.660	0.578	0.667	0.624	0.915	0.866
	Random Forest	0.342	0.910	0.781	0.765	0.439	0.646	0.542	0.930	0.869
	NITROGEN	0.513	0.778	0.748	0.550	0.557	0.589	0.566	0.918	0.874
	NAIM	0.390	0.814	0.735	0.615	0.496	0.565	0.551	0.910	0.856
ADNI (Test)	XGBoost	0.516	0.854	0.799	0.680	0.541	0.654	0.523	0.928	0.859
	LightGBM	0.532	0.833	0.781	0.644	0.573	0.647	0.562	0.919	0.859
	Random Forest	0.332	0.918	0.788	0.779	0.404	0.643	0.460	0.931	0.856
	NITROGEN	0.478	0.809	0.775	0.631	0.535	0.619	0.521	0.926	0.863
	NAIM	0.382	0.825	0.744	0.642	0.423	0.547	0.411	0.926	0.830
OASIS-3	LightGBM	0.223	0.895	0.677	0.449	0.454	0.429	0.477	0.787	0.684
	Random Forest	0.092	0.951	0.684	0.755	0.139	0.361	0.173	0.965	0.740
	NAIM	0.136	0.937	0.686	0.245	0.648	0.441	0.613	0.489	0.656
	NITROGEN	0.201	0.899	0.680	0.531	0.323	0.378	0.374	0.899	0.691
	XGBoost	0.154	0.911	0.653	0.388	0.406	0.380	0.475	0.771	0.690
AIBL	NAIM	0.332	0.873	0.750	0.745	0.351	0.555	0.361	0.981	0.881
	Random Forest	0.304	0.888	0.762	0.777	0.311	0.537	0.323	0.993	0.886
	LightGBM	0.447	0.805	0.724	0.630	0.460	0.557	0.400	0.979	0.896
	XGBoost	0.470	0.785	0.728	0.598	0.467	0.543	0.342	0.984	0.905
	NITROGEN	0.291	0.873	0.745	0.723	0.312	0.505	0.374	0.984	0.907

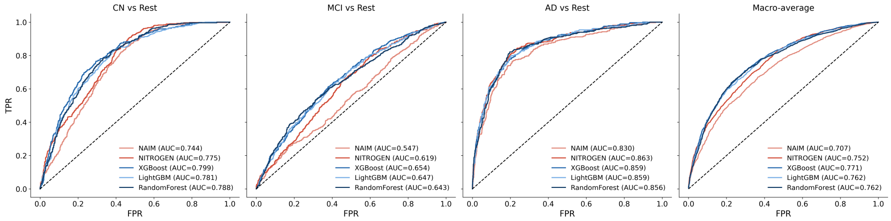

(a) ADNI test subset

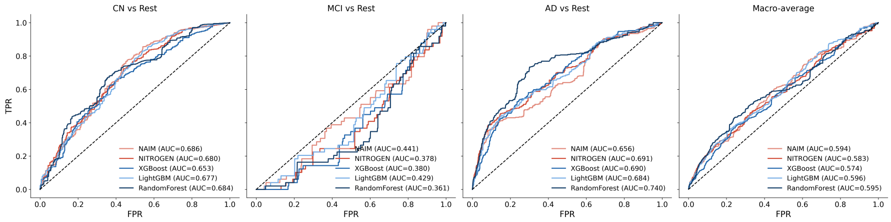

(b) OASIS-3 test set

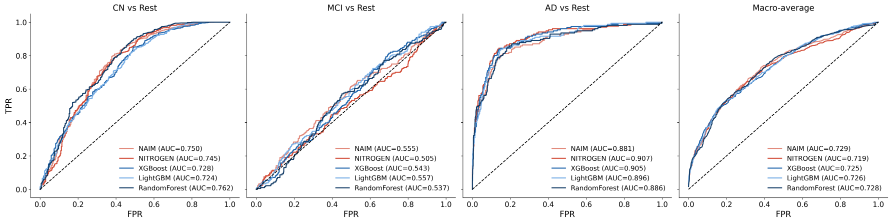

(c) AIBL test set

Fig. A1: **Classification performance in multi-class cognitive group classification.** ROC curves (One vs. Rest) for multi-class classification on (a) ADNI test set, (b) OASIS-3 and (c) AIBL. For each test set, the plots represent the ROC curves for the CN, MCI, AD vs. the rest and macro average from left to right. AUC values are reported in the legends. All models trained on ADNI.

Table A6: Performance of models trained on ADNI for regression of MMSE and CDR-SB scores, evaluated across datasets. ADNI comprised 7,858 samples (CN: 2,497, MCI: 3,583, AD: 1,778) divided into 60/20/20 train/validation/test splits. OASIS-3 (CN: 2,228, MCI: 49, AD: 398) and AIBL (CN: 947, MCI: 184, AD: 155) were used as held-out external validation cohorts. Within each split, models are sorted by MMSE R^2. Seed = 123.

		MMSE					CDR-SB				
Data/Split	Model	MSE	MAE	R^2	r	p	MSE	MAE	R^2	r	p
ADNI (Train)	XGBoost	0.000	0.000	1.000	1.000	0	0.000	0.000	1.000	1.000	0
	Random Forest	3.075	1.159	0.788	0.918	0	1.626	0.828	0.736	0.892	0
	NITROGEN	6.721	1.843	0.536	0.733	0	3.079	1.185	0.500	0.713	0
	NAIM	7.151	1.876	0.507	0.714	0	3.121	1.223	0.493	0.703	0
ADNI (Val)	NITROGEN	7.745	2.027	0.446	0.670	1.18e−203	3.586	1.309	0.415	0.648	1.10e−186
	Random Forest	8.015	2.076	0.427	0.661	6.46e−197	3.633	1.363	0.407	0.650	4.09e−188
	NAIM	8.389	2.081	0.400	0.636	5.69e−178	3.815	1.384	0.377	0.616	1.41e−163
	XGBoost	9.049	2.239	0.353	0.617	2.68e−164	3.811	1.384	0.378	0.632	3.58e−175
ADNI (Test)	NAIM	8.206	2.007	0.383	0.627	1.58e−174	3.486	1.337	0.402	0.635	9.47e−181
	NITROGEN	8.216	1.999	0.382	0.621	1.67e−170	3.517	1.302	0.397	0.641	3.19e−185
	Random Forest	8.417	2.069	0.367	0.610	8.87e−163	3.578	1.352	0.386	0.633	6.63e−179
	XGBoost	9.450	2.205	0.289	0.569	2.41e−137	3.853	1.409	0.339	0.604	1.10e−158
AIBL	NITROGEN	8.255	1.727	0.384	0.664	2.67e−164	1.551	0.901	-8.771	0.603	2.87e−128
	NAIM	8.571	1.775	0.360	0.635	4.06e−146	2.402	1.246	-14.136	0.554	1.87e−104
	Random Forest	8.744	1.767	0.347	0.666	2.45e−165	1.642	1.095	-9.344	0.656	2.91e−159
	XGBoost	9.196	1.871	0.313	0.579	3.39e−116	2.127	1.076	-12.405	0.568	8.88e−111
OASIS-3	Random Forest	9.738	2.426	-0.637	0.285	3.02e−51	4.797	1.879	-62.886	0.284	6.31e−51
	NAIM	11.928	2.770	-1.005	0.303	5.02e−58	7.896	2.456	-104.163	0.247	2.24e−38
	XGBoost	13.555	2.270	-1.278	0.278	8.68e−49	7.253	1.692	-95.592	0.263	1.09e−43
	NITROGEN	16.326	3.004	-1.744	0.245	5.36e−38	5.725	2.079	-75.252	0.255	4.77e−41

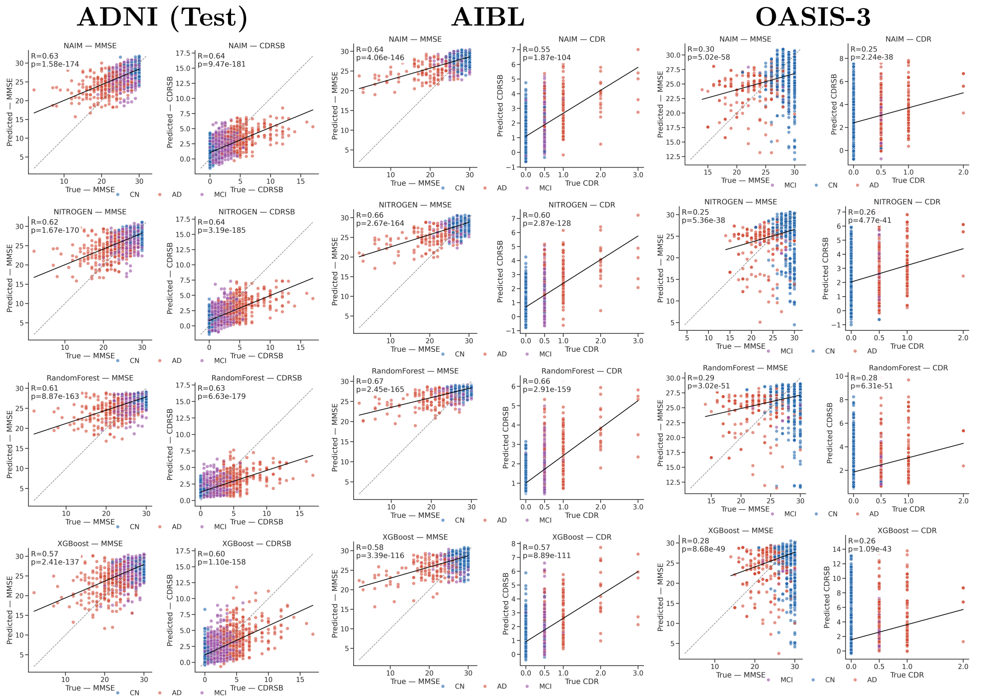

Fig. A2: **Regression performance across models and datasets.** Predicted versus observed values for MMSE and CDR-SB scores across test datasets (columns: ADNI, AIBL, OASIS) and models (rows). Each point represents an individual, coloured by diagnostic group (CN: green, MCI: purple, AD: red). The dashed line indicates perfect regression, and the solid line shows the least-squares fit. Pearson correlation coefficients (r) are reported in each panel. Models were trained on ADNI and evaluated on internal (ADNI) and external (AIBL, OASIS) cohorts.

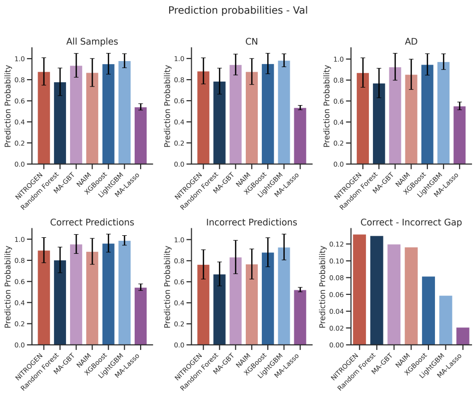

(a) ADNI validation subset

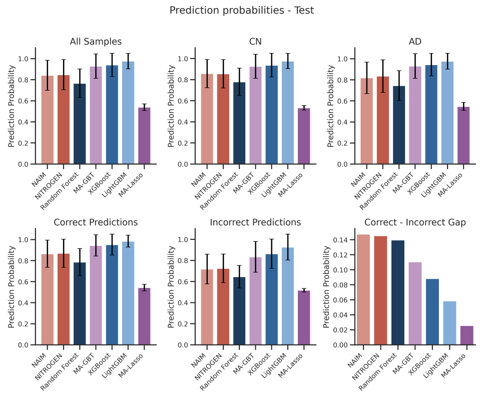

(b) ADNI test subset

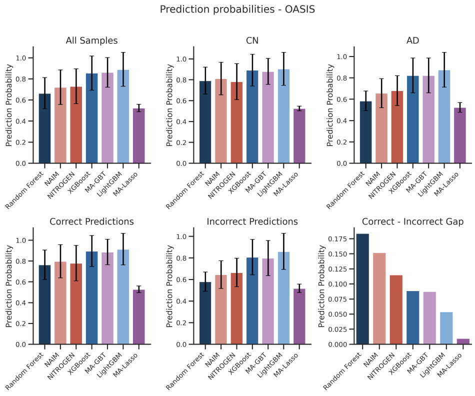

(c) OASIS-3 test set

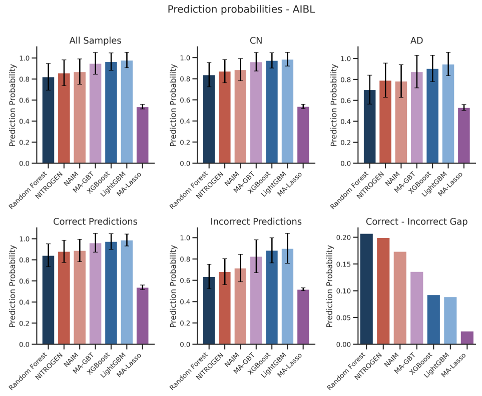

(d) AIBL test set

Fig. A3: **Predicted class probabilities across internal and external datasets.** Bar plots show mean predicted class probabilities across models, with error bars representing one standard deviation across samples (not shown for the correct–incorrect confidence gap, which reflects a derived difference between two conditions). Within each dataset, results are displayed for all samples, stratified by predicted class (CN and AD), by correctness of prediction (correct vs. incorrect), and by the correct–incorrect confidence gap. The confidence gap is defined as the difference between the mean predicted probability of correctly classified samples and that of incorrectly classified samples.

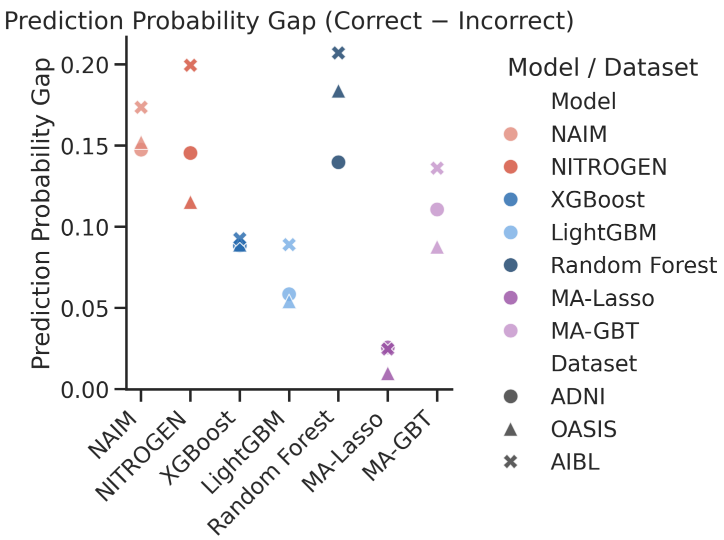

Fig. A4: **Prediction probabilities gap across models and test datasets.** Scatter plot summarising the prediction confidence gap, defined as the difference between the mean predicted probability of correctly classified samples and that of incorrectly classified samples (correct − incorrect). Each point represents a model–dataset pair. Colours indicate the classification model, and marker shapes denote the evaluation dataset (ADNI, OASIS-3, AIBL). Higher values indicate stronger separation between correct and incorrect predictions, reflecting improved confidence discrimination.

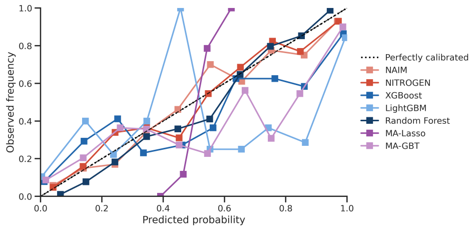

(a) ADNI validation set

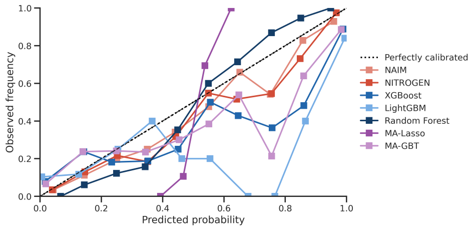

(b) ADNI test set

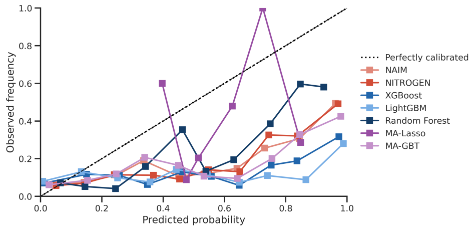

(c) External OASIS-3 cohort

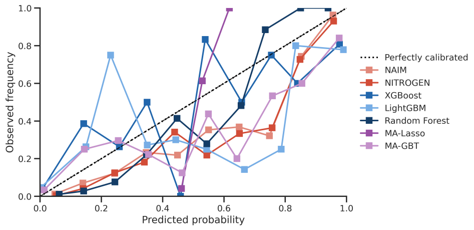

(d) External AIBL cohort

Fig. A5: **Calibration performance across internal and external datasets.** Reliability diagrams showing model calibration for the positive class (AD). The mean predicted probability within each bin (x-axis) is plotted against the observed fraction of positives (y-axis). Colours correspond to different classification models. The dotted black diagonal represents perfect calibration. Models were trained on ADNI and evaluated on the validation set (a), ADNI test set (b), OASIS-3 (c), and AIBL (d).

Table A7: Sufficiency analysis using the identified features (APOEε4 and left temporal pole cortical thickness). Performance is reported for three input configurations: (i) full multimodal input (Base), (ii) only the identified MRI feature (LH_Limbic_TempPole_3) with all other MRI modalities removed (set to NA) (MRI), and (iii) only the MRI feature with all others masked (ALL). Metrics include accuracy (Acc.), balanced accuracy (Bal. Acc.), specificity (Spec.), sensitivity (Sens.), and area under the ROC curve (AUC). Seed = 123.

Dataset	Modality	Model	Acc.	Bal. Acc.	Spec.	Sens.	AUC
ADNI (Test)	Base	NAIM	0.87	0.86	0.88	0.85	0.91
		NITROGEN	0.85	0.84	0.86	0.82	0.91
		XGBoost	0.87	0.86	0.88	0.84	0.91
		LightGBM	0.85	0.84	0.86	0.82	0.92
		Random Forest	0.85	0.84	0.88	0.81	0.92
		MA-Lasso	0.82	0.83	0.80	0.86	0.91
		MA-GBT	0.83	0.82	0.85	0.79	0.90
	MRI	NAIM	0.53	0.63	0.30	0.96	0.76
		NITROGEN	0.58	0.66	0.41	0.91	0.77
		XGBoost	0.65	0.71	0.54	0.87	0.79
		LightGBM	0.70	0.72	0.65	0.80	0.78
		Random Forest	0.51	0.61	0.30	0.92	0.77
		MA-Lasso	0.57	0.63	0.45	0.81	0.77
		MA-GBT	0.75	0.71	0.84	0.59	0.80
	ALL	NAIM	0.34	0.50	0.00	1.00	0.77
		NITROGEN	0.34	0.50	0.00	1.00	0.77
		XGBoost	0.53	0.62	0.33	0.92	0.76
		LightGBM	0.66	0.50	1.00	0.00	0.76
		Random Forest	0.34	0.50	0.00	1.00	0.57
		MA-Lasso	0.72	0.71	0.76	0.66	0.77
		MA-GBT	0.66	0.50	1.00	0.00	0.76
AIBL	Base	NAIM	0.90	0.78	0.94	0.62	0.92
		NITROGEN	0.90	0.81	0.93	0.69	0.96
		XGBoost	0.93	0.79	0.98	0.60	0.94
		LightGBM	0.93	0.80	0.98	0.62	0.94
		Random Forest	0.90	0.75	0.95	0.55	0.92
		MA-Lasso	0.92	0.88	0.94	0.83	0.97
		MA-GBT	0.93	0.81	0.97	0.64	0.93
	MRI	NAIM	0.38	0.63	0.30	0.95	0.81
		NITROGEN	0.57	0.71	0.53	0.88	0.79
		XGBoost	0.81	0.60	0.87	0.33	0.73
		LightGBM	0.71	0.66	0.72	0.60	0.70
		Random Forest	0.15	0.47	0.05	0.88	0.57
		MA-Lasso	0.74	0.75	0.74	0.76	0.81
		MA-GBT	0.87	0.64	0.95	0.33	0.79
	ALL	NAIM	0.12	0.50	0.00	1.00	0.83
		NITROGEN	0.12	0.50	0.00	1.00	0.86
		XGBoost	0.40	0.65	0.32	0.98	0.86
		LightGBM	0.88	0.50	1.00	0.00	0.86
		Random Forest	0.12	0.50	0.00	1.00	0.41
		MA-Lasso	0.82	0.81	0.83	0.79	0.86
		MA-GBT	0.88	0.50	1.00	0.00	0.87
OASIS-3	Base	NAIM	0.49	0.60	0.44	0.76	0.68
		NITROGEN	0.54	0.65	0.49	0.81	0.72
		XGBoost	0.51	0.60	0.47	0.73	0.69
		LightGBM	0.52	0.57	0.49	0.66	0.67
		Random Forest	0.43	0.56	0.38	0.74	0.64
		MA-Lasso	0.55	0.61	0.52	0.70	0.70
		MA-GBT	0.68	0.65	0.69	0.60	0.72
	MRI	NAIM	0.28	0.53	0.18	0.88	0.65
		NITROGEN	0.39	0.59	0.30	0.87	0.65
		XGBoost	0.33	0.58	0.23	0.93	0.65
		LightGBM	0.38	0.54	0.31	0.77	0.62
		Random Forest	0.19	0.51	0.05	0.97	0.59
		MA-Lasso	0.46	0.57	0.42	0.72	0.60
		MA-GBT	0.69	0.64	0.71	0.56	0.69
	ALL	NAIM	0.15	0.50	0.00	1.00	0.62
		NITROGEN	0.15	0.50	0.00	1.00	0.55
		XGBoost	0.28	0.56	0.16	0.96	0.60
		LightGBM	0.85	0.50	1.00	0.00	0.56
		Random Forest	0.15	0.50	0.00	1.00	0.51
		MA-Lasso	0.76	0.57	0.84	0.30	0.57
		MA-GBT	0.85	0.50	1.00	0.00	0.54

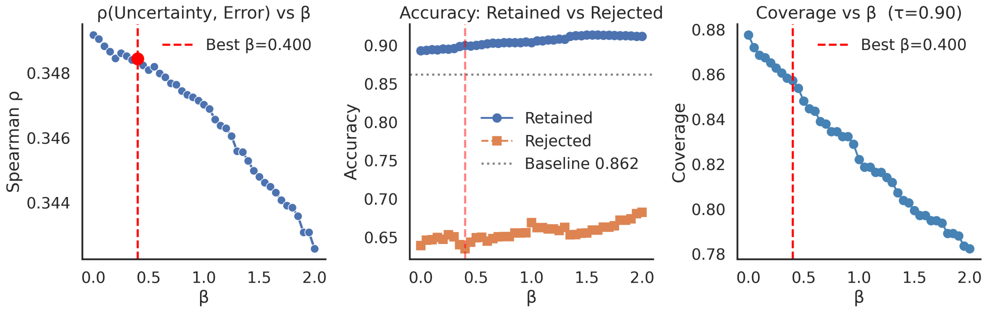

Fig. A6: Grid search results for the sensitivity parameter β at fixed uncertainty threshold $\tau = 0.900$. **(Left)** Spearman rank correlation ρ between adjusted uncertainty and prediction errors as a function of β, used as the primary optimisation criterion. The optimal value $\beta^* = 0.400$ is indicated by the vertical red dashed line. **(Centre)** Accuracy on retained and rejected samples as a function of β, with the baseline accuracy on the full dataset shown as a horizontal dotted line. A well-calibrated β should increase the gap between retained and rejected accuracy. **(Right)** Coverage (fraction of samples retained) as a function of β, showing how the missingness adjustment progressively shifts samples above the fixed threshold τ as β increases. The optimal configuration ($\beta^* = 0.400$, $\tau = 0.900$) retains 85.7% of validation samples (756/882), improving accuracy from 0.8617 to 0.8995 ($\Delta = +0.0378$, $p < 0.001$ by permutation test), with rejected samples exhibiting substantially lower accuracy (0.6349).
